# A Metabolomic Investigation of the Effect of Eosin B on Game-tocyte of *Plasmodium falciparum Using* 1HNMR Spectroscopy

**Published:** 2019

**Authors:** Alireza SADEGHI TAFRESHI, Zahra ZAMANI, Marjan SABBAGHIAN, Ramezan Ali KHAVARI-NEJAD, Mohammad ARJMAND, Sedigheh SADEGHI, Maryam MOHAMMADI

**Affiliations:** 1. Department of Biochemistry, Science and Research Branch, Islamic Azad University, Tehran, Iran; 2. Department of Biochemistry, Pasteur Institute of Iran, Tehran, Iran; 3. Department of Andrology at Reproductive Biomedicine Research Center, Royan Institute for Reproductive Biomedicine, ACECR, Tehran, Iran

**Keywords:** Malaria, *Plasmodium falciparum*, Gametocyte, Eosin B, Lactate dehydrogenase, Metabolomics, ^1^HNMR

## Abstract

**Background:**

Recently eosin B was shown to have an effect on the asexual stage of *Plasmodium falciparum* and in this study, its activity against gametocytes and changes in the culture medium metabolites were investigated using an^1^HNMR-based metabolomics approach.

**Methods:**

In the Biochemistry Department of Pasteur Institute of Iran in 2017, parasites were cultured and gametocytogenesis induced by heparin and 5% hematocrit. Sexual stage parasites were tested by eosin B in 90 well plates and IC_50_ determined using Lactate Dehydrogenase assay. Gametocytes were treated by IC_50_ dose of eosin B and the medium collected in the two groups: with eosin B and controls and sent for ^1^HNMR spectroscopy. The spectra were analyzed on MATLAB interface and the altered metabolites in the culture medium and eosin-affected biochemical pathways were identified by Human Metabolome Database and Metabo-analyst website.

**Results:**

The results revealed eosin B had an effective gametocytocidal activity against *P. falciparum*. The significant metabolites changed in the medium were thia-mine, Asp, Asn, Tyr, Lys, Ala, Phenylpyruvic acid, NAD^+^ and lipids. The main pathways identified were aminoacyl-tRNA biosynthesis, Phenylalanine, tyrosine and tryptophan biosynthesis, Alanine, aspartate and glutamate metabolism, Phenylala-nine metabolism, Nicotinate and nicotinamide metabolism, and lysine degradation.

**Conclusion:**

Eosin B exhibited substantial gametocytocidal activity and affected important drug targets in the *Plasmodium*.

## Introduction

Malaria is a major health and economic problem globally and is a preventable and treatable parasitic disease caused by *Plasmodium* species. The WHO site reports the death of 445000 people and 216 million cases of malaria worldwide despite the 2.7 billion dollars invested in 2016 (1). To date, it is only the artemisinin-based combination therapies (ACTs) that rapidly reduce both asexual and gametocyte stages of *P. falciparum* (2).

However, information about the malaria parasite sexual stage seems to be limited due to the fact that its in vitro culture is still technically challenging. There seems to be almost no standard high thorough put screening assay for its drug testing as it does not interfere directly in the signs and symptoms of the disease. Hence any drug that can inhibit the transmission of gametocytes to the mosquito can be useful in elimination of malaria (3).

Eosin B, which is a laboratory stain is effective on the asexual blood stage of *P. falciparum invitro* (4), as well as on *P. berghei*, murine malaria (5). In our recent studies, eosin B showed a significant synergistic effect in combination with other traditional drugs (artemisinin, chloroquine, sulfadoxine-pyrimethamine) on the parasite asexual stage (article in press). Methylene blue, another laboratory stain has been used as an antimalarial in synergy with chloroquine and has shown gametocytocidal properties (6). The safety of eosin B has been approved by FDA and is widely used in cosmetics (7).

Metabolomics is the complete and simultaneous detection of metabolites using high throughput technology and their changes in response to factors such as diet, drugs, environmental stimuli and genetic variations. Spectroscopy methods such as Nuclear Magnetic Resonance (NMR) and Mass spectroscopy (MS) methods are utilized to provide structural information about the different metabolites (8). NMR though not as sensitive as MS is a non-destructive and highly reproducible spectroscopic technique, requires minimal sample preparation and can provide valuable metabolomic information (9).

As the metabolomics studies provide a large volume of data, chemometrics is used to accurately process the data using different statistical analysis like Principle component analysis (PCA) and partial least squares discriminant analysis (PLS-DA). PCA is a statistical technique that shows the best linear transformation for a group of data points which exhibits the sample properties along with the coordinate (i.e. principal) axes, hence metabolomics data can be clustered, plotted and visualized based on the linear combinations of their shared features. PLS-DA likewise classifies and analyzes very large data sets containing a low number of samples (10).

In this study, the gametocytocidal activity of the eosin B was tested on *P. falciparum.* For this purpose, the effect of the drug on gametocytes was tested by microscopy and further evaluation of the efficacy of the drug was carried out using Malstat assay, an enzymatic test based on parasite lactate dehydrogenase. A metabolomics study was carried out to investigate the metabolic profile of the drug-treated gametocyte media and untreated controls using ^1^HNMR spectroscopy and chemometrics analysis. After identification of the differentiating metabolites by Human Metabolome Database (HMDB). The major biochemical pathways affected by the drug were resolved by the Metabo-Analyst website and the biochemical mechanisms of the effect of eosin B on gametocytes were determined.

## Materials and Methods

### Plasmodium falciparum gametocyte culture

*P. falciparum* parasites were cultured in human RBCs with 2% hematocrit at 35.5 °C in complete culture medium [containing RPMI 1640 medium (Sigma-Aldrich) supplemented with 25 mM HEPES (Sigma-Aldrich), 0.2% D-glucose (Sigma-Aldrich), 200 μM hypoxanthine (Sigma-Aldrich), 0.2% sodium bicarbonate, 24 μg/ml gentamicin (Invitrogen)] and 10% human serum (preferably pooled serum) or 0.5% Albumax II with mixed gas consisting of 90% N_2_/5% O_2_/3% CO_2_ in Biochemistry Department, Pasteur Institute of Iran in 2017 (11). Culture medium (pre-warmed to 35.5 °C) was replaced daily and Giemsa-stained blood smears were microscopically examined to monitor parasite proliferation. Synchronization of trophozoite stage of parasites was carried out using 5% sorbitol (Sigma-Aldrich). Gametocytogenesis was induced by adding 5% blood (type O+) and warmed to 37 °C. The culture medium was replaced daily with heparin (20 U/ml) keeping one-third of the consumed medium. Asexual and sexual stages parasitemia were determined by counting 10,000 cells in the Giemsa-stained thin smears until the 14^th^ day (12). Safety precautions were considered (13).

### Test of eosin B on gametocytes in vitro

Overall, 20 μl of the above culture containing 1%–2% gametocytes was added with 100 μl of serial dilutions of the drug (to all except the control wells which contained the culture but no drugs). The experiment was carried out in triplicate and the plates were placed at 37 °C for 48 h inside the candle jar.

### Plasmodium LDH assay (pLDH)

Overall, 100 μl/well of eosin B were added in different serial dilutions in triplicate with 100 μl/well of stage IV and V gametocyte cultures to achieve a final 1%–2% gametocytaemia and 2% haematocrit in 96-well plates. The plates were incubated at 37 °C in candle jar conditions for 48 h. The viability of the gametocytes was obtained by measuring the activity of pLDH spectrophotometrically according to a modified version of the method of Makler and Hinrichs (14). Malstat reagent was prepared (0.21% v/v Triton-100; 222 mM L-(+)- lactic acid; 54.5 mMTris; 0.166 mM 3-acetylpyridine adenine dinucleotide (APAD; Sigma-Aldrich); adjusted to pH 9 with 1 M NaOH) and 100 μl was transferred into a clean 96-well plate and 20 μl parasite suspension/well was added to it, followed by the addition of 25 μl PES/NBT (1.96 mM nitro blue tetrazolium chloride NBT; 0.239 mMphenazineetho sulfate PES). Spectrophotometric absorbance was measured with a Microplate Spectrophotometer (BioTekPowerWave XS) at 650 nm (15).The results were expressed as percentage viability compared with untreated controls and were calculated by the following formula (16):

$$100\times ({\text{OD}}_{\text{treated}\;\text{sample}}-\mu_{\text{c}-})/\left(\mu_{\text{c}+}-\mu_{\text{c}-}\right)$$

μ (means), c+ (OD control gametocytes) and c- (OD blank uninfected RBCs)

### Statistical Data Analysis

The percentage of viability obtained from pLDH assay was plotted as a function of drug concentration.

### ^1^HNMR acquisition

Two groups of late-stage gametocytes of four flasks each were treated with eosin B and the other untreated as controls. The media was separated from the infected RBCs and centrifuged at 3000 × g for 10 min to remove the parasites and the used media were analyzed by ^1^HNMR spectroscopy. A standard one-dimensional protocol was applied and data was acquired by CPMG spin-echo ^1^HNMR pulse programmer for 128 scans (after eight dummy scans), which included water irradiation during the recycle delay, set at 2 sec at 500 MHZ NMR spectrometer Bruker was used for spectroscopy.

### Chemometrics data analysis

To analyze the data the Prometab programme (17) software was applied in the MATLAB software, the raw NMR spectrum was converted into matrices for multivariate analysis by the statistical analysis option on Metabo-Analyst software (18). The spectra were normalized, the data were analyzed by Principle component analysis to obtain the score plots and loading plots and Partial Least Squares-Discriminant Analysis *(PLS-DA)* from which the chemical shift of differentiated metabolites were obtained and identified using the Human Metabolism Database (HMDB) (19), in both the experimental and control groups. The Metabo-Analyst pathway analysis option (18) was used to identify the changed metabolic pathways. The Kyoto Encyclopedia of Genes and Genomes (KEGG) database (20) was used to study the altered pathways related to the metabolites.

## Results

**Fig. 1:**
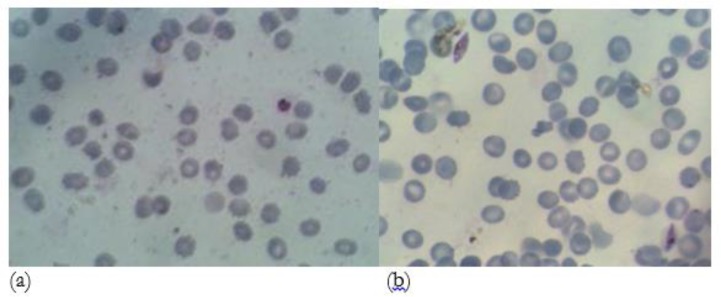
Cultivation of asexual stages and gametocytes. (a) asexual parasite (ring stage), (b) gametocytes

Asexual parasitemia and gametocytemia during the 14 d period were determined by manual counting of Giemsa-stained culture smears (Fig. 1).

**Fig. 2:**
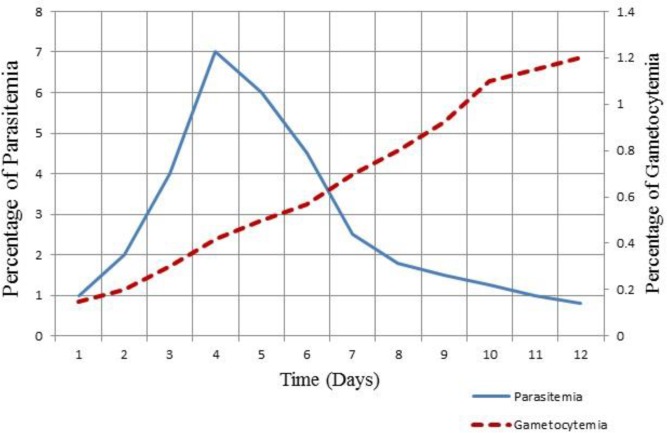
Percentage of parasitemia and gametocytemia during gametocyte culturing

**Fig. 3:**
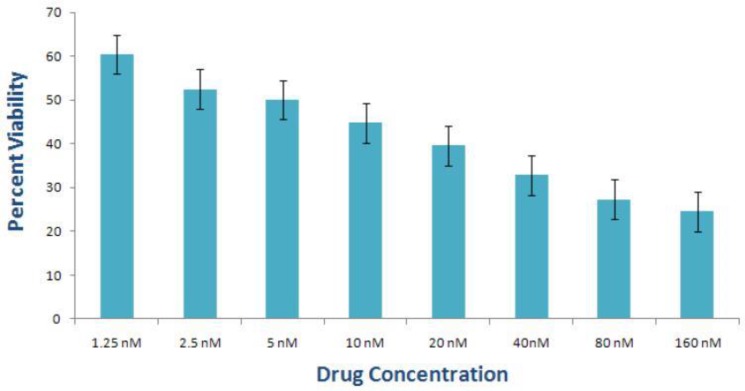
LDH assay of eosin B-treated *P. falciparum* gametocytes

Asexual parasitemiaon day 4 reached its peak and then gradually diminished. The gametocytemia increased over time and reached the maximum on day 16 (Fig. 2). Gametocyte viability was evaluated by the PfLDH assay (Fig. 3).

**Fig. 4:**
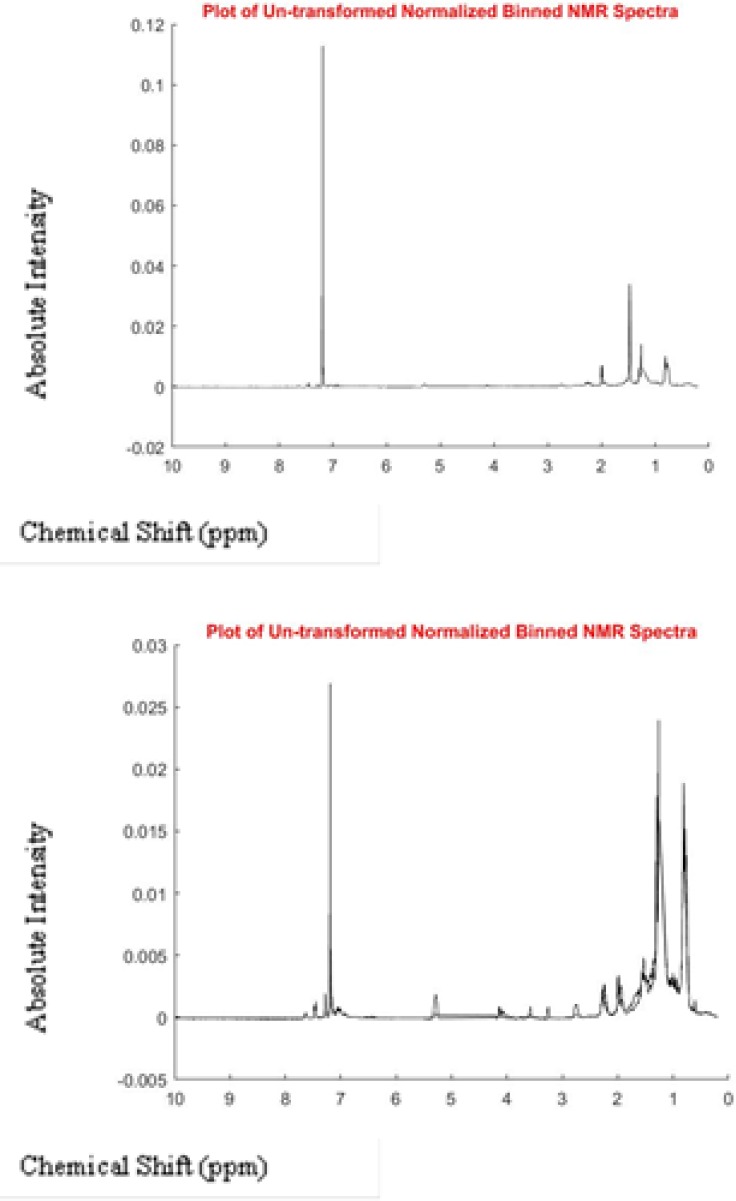
Plot of Untransformed Normalized Binned NMR Spectra. (a) Control, (b) Drug test X axis is chemical shift in ppm and y axis absorption intensity

The NMR spectra of both groups (control and test) are shown in Fig. 4a and 4b.

**Fig. 5:**
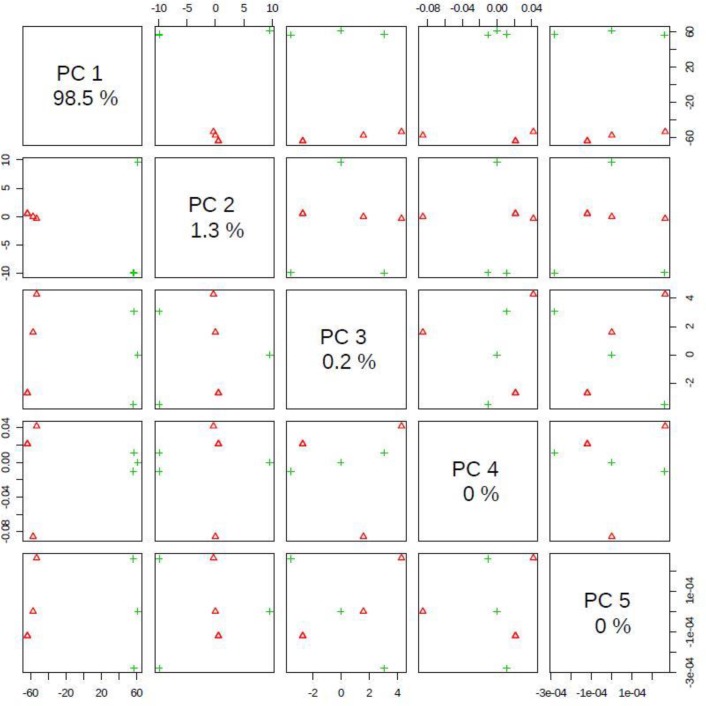
Pairwise score plots between the selected PCs. The explained variance of each PC is shown in the corresponding diagonal cell

Fig. 5 shows the score plot of PCA with PC1 showing 98% separation between the two groups.

**Fig. 6:**
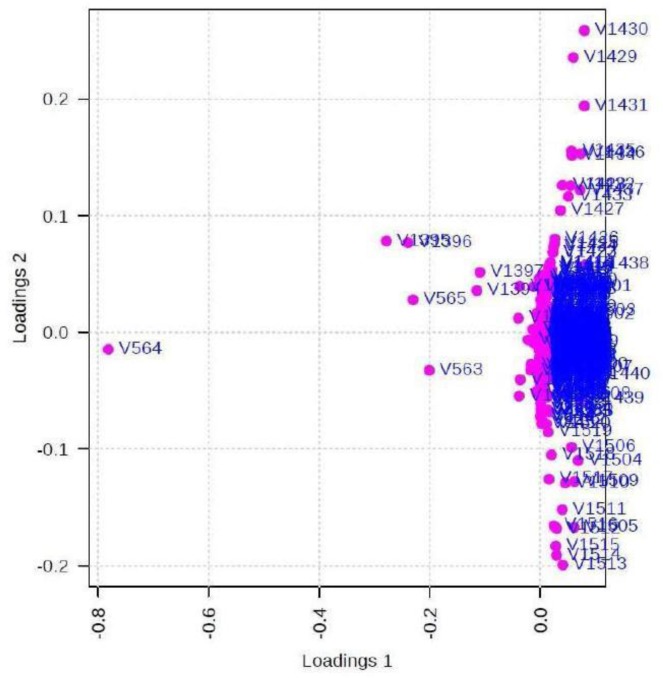
Loadings plot for the selected PCs

The loading plot is shown in Fig. 6 with overlapping chemical shifts which are the common metabolites in the two groups but the differentiating chemical shifts are clearly depicted.

**Fig. 7:**
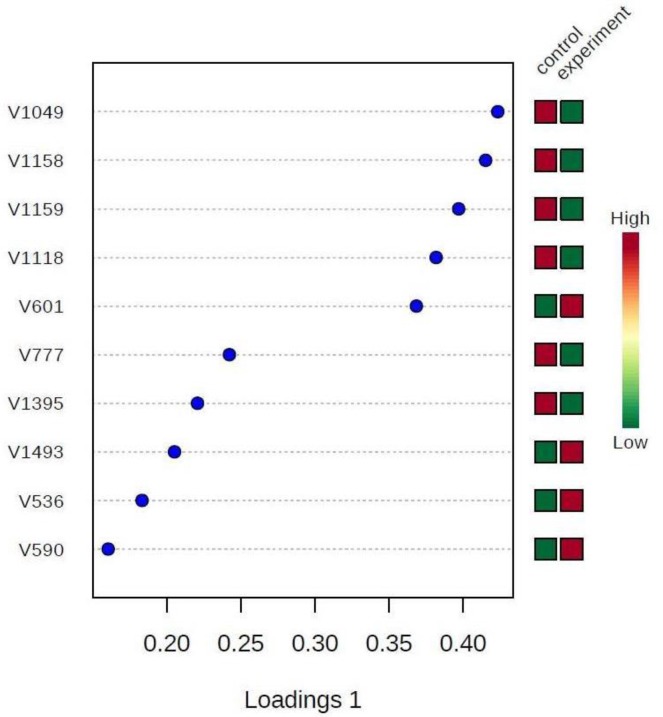
Levels of differentiating metabolites identified by PLS-DA

Fig. 6 displays the loading plot with the superimposed chemical shifts showing the common metabolites and the others depicting differentiating metabolites. The levels of differentiating metabolites are identified PLS-DA in Fig. 7.

The metabolites identified by Human Metabolome Database are represented in Table 1 and the pathways and their *P*-values are in Table 2.

**Table 1:** Significant altered metabolites by treatment with eosin B

***Number***		***Chemical shift***	***Metabolite***	***HMDB ID***
1	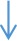	3.2075	Thiamine	HMDB00235
2	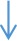	2.6625–2.6675	L-Aspartic acid	HMDB00191
3	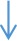	2.8625	L-Aspargine	HMDB00168
4	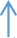	7.0075	L-tyrosine	HMDB00158
5	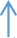	3.0225	L-lysine	HMDB00182
6	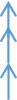	6.1275	NAD	HMDB00902
7	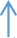	1.4775	L-Alanine	HMDB00161
8	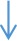	0.8675	TG	HMDB05356
9	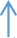	7.3325	Phenylpyruvic acid	HMDB00205

**Table 2:** Results from Pathway Analysis

***Pathway name***	***Total***	***Expected***	***Hits***	***Raw p***
Aminoacyl-tRNA biosynthesis	46	0.82	5	4.06E-04
Phenylalanine, tyrosine and tryptophan biosynthesis	6	0.11	2	4.03E-03
Alanine, aspartate and glutamate metabolism	12	0.21	2	1.68E-02
Phenylalanine metabolisn	3	0.05	1	5.26E-02
Nicotinate and nicotinate metabolism	7	0.12	1	1.19E-01
Lysine degradation	12	0.21	1	1.96E-01

**Fig. 8:**
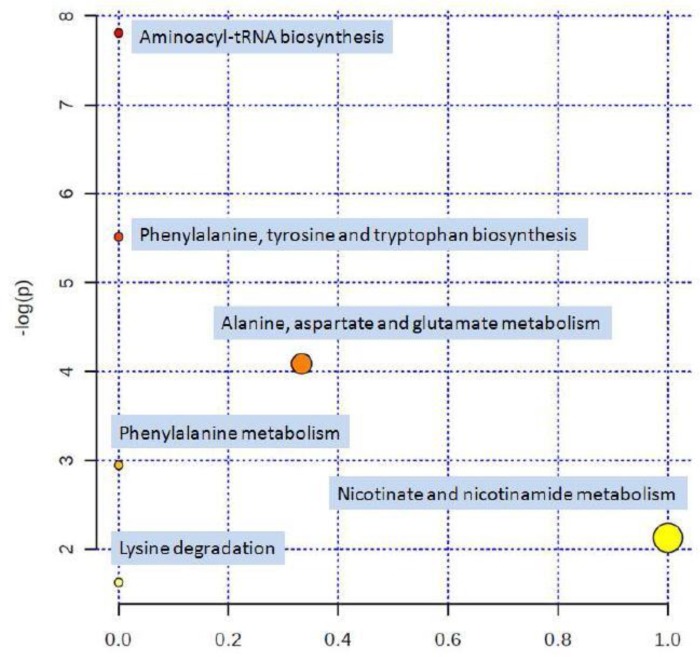
Summary of Pathway Analysis. Circles higher, bigger and closer to the x-axis are more significant

The metabolites were entered into the Metabo Analyst database and the pathways analysis in Fig. 8 showed the pathways affected by eosin B.

## Discussion

Gametocytocidal drugs are not very common, at present, there are only two known for this activity; primaquine and tafenoquine which inhibit gametocytes belonging to all the species of the human *Plasmodium*. However, late-stage gametocytes are not killed by artemisinin but seem to be indirectly inhibited due to the prompt clearance of the asexual stage and immature gametocytes from the bloodstream (21). There are clear reports of prima-quine stopping transmission even before complete removal of the parasite from the patient's blood but its extensive use in malaria eradication is prevented due to hemolytic toxicity caused in patients with deficiency of glucose-6-phosphate dehydrogenase common in some endemic countries (22).

**Fig. 9:**
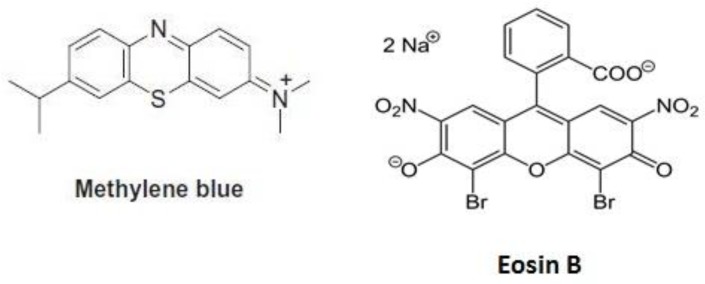
Chemical structure of laboratory dyes used as antimalarial drugs

Methylene blue and eosin B, both of which are common laboratory dyes can effectively reduce gametocytemia in *P. falciparum* (Fig. 9). The IC_50_ of eosin B on *P. falciparum* 3D7 gametocytes is 23 nM near that of methylene blue (IC_50_ 12 nM), whereas hydroxychloroquine IC_50_ is 22.78 nM and that of artesunate 102.3 nM and primaquine is 15μmol (23, 24). In addition, metabolomics studies on the culture medium demonstrate the effect of this compound on parasite metabolism (25). Since few compounds have significant effects on gametocytes, this activity of eosin B is very important and makes it a promising malaria transmission-blocking agent, but further studies are needed for this purpose.

As gametocytes comprise 5% of the *Plasmodium* culture and metabolic studies require a great amount of protein, it was decided to analyze the culture medium by ^1^HNMR for any changes in the nutrients and correlate them to the changes in the pathways of the *P. falciparum*. Time variations in the culture medium of gametocytes were studied earlier and found changes in the amino acid levels in the different stages of Plasmodium growth (26). In this study too, certain amino acids, thia-mine, NAD and triglycerides exhibited metabolite level changes as shown in Table 1. L-aspartic acid, aspargine and tyrosine participate in pyrimidine and purine biosynthesis directly and in the TCA cycle indirectly as shown in supplementary data. The thiamine detected participates in glycolysis, purine metabolism and metabolism of tyrosine and glycine and thus pyrimidine and purine biosyn-thesis and the TCA cycle are affected by Eosin B.

The pathway analysis shows the role of bio-synthesis of aminoacyl t-RNA on the production of L-alanyl-tRNA, L-aspartyl-tRNA, L-asparaginyl-tRNA, L-lysyl-tRNA and L-tyrosyl-tRNA from their consecutive amino acids and interruption of protein synthesis in the parasite. The formation of amino-acyl t-RNA requires important enzymes namely aminoacyl-t-RNA synthetase who secrystalline structures have been identified and are thought to be suitable drug targets for organisms such as *P. falciparum* (27). A feature of protein translation that makes it an acceptable drug target is the vast evolutionary distance between parasites and the human host as many pathways are not shared between the two. Many apicomplexan parasites carry out this process in the apicoplast not seen in mammalian cells and hence, these enzymes can be described as suitable drug targets (28).

The next pathway in the gametocytes to be affected by eosin B is the biosynthesis of phenylalanine, tyrosine and tryptophan with phenyl-pyruvate and tyrosine as the metabolites detected. As phenylalanine, tyrosine and tryptophan are aromatic amino acids and organisms such as plants, fungi and some protozoan parasites have a pathway named shikimate pathway to produce these aromatic molecules from simple products of carbohydrate metabolism. The precursor is named chorismate which metabolizes to para-aminobenzoate, ubiquinone and aromatic amino acids. The absence of shikimate pathway in mammals has made it a highly distinctive target in chemo-therapy against malaria (29). Eosin B, as mentioned above, has been effective in the biosynthesis of aromatic amino acids and can thus act a gametocytocidal agent.

The alanine, aspartate and glutamate metabolism pathway showed a change in the respective amino acids. L-alanine is changed to pyruvate and enters the glycolysis cycle. This cycle is indirectly related to the pyrimidine cycle. In previous studies, the amount of glutamine supplied by this metabolic pathway increases in the brains of cerebral malaria-infected mice, while glutamine decreases in serum. There have been unconfirmed early reports that the parasite has the ability to synthesis alanine, aspartate, and glutamate after fixing carbon dioxide but amino acid consumption from the erythrocytes uptake is proven (30).

Tyrosine and phenyl-pyruvate metabolites also have been altered in the pathway of phenylalanine metabolism. There are reports of valine, alanine and phenylalanine exported from the infected RBC into the medium (31). However, regulation of levels of phenylalanine are essential for proper functioning of the cells. Though phenylalanine hydroxylase is absent in *Plasmodia*, three different assemblies of genes are present for phenylalanine tRNA-synthetase in the three different compartments, the mitochondria, the apicoplast and the cytoplasm. Hence, the phenylalanine cycle is important as a drug target (32).

Nicotinamide adenine dinucleotide (NAD^+^) is a vital metabolite used as a redox cofactor and substrate for enzymes in many cellular processes. Red blood cells infected with the malaria parasite *P. falciparum* show higher NAD^+^ levels than uninfected ones, but its mechanism is not yet understood. A metabolomics study using mass spectrometry verified that *P. falciparum* does not synthesize NAD^+^ de novo and is dependent on the consumption of exogenous niacin (33). The research provides novel therapeutic targets for new antimalarial drugs and reveals the importance of the NAD^+^ metabolic pathway of the parasite.

Lysine degradation is also involved with eosin B due to the metabolite lysine and in the *Plasmodium* lysine is obtained from digestion of hemoglobin digestion or extracellular space implicated in protein synthesis. Pipecolate, but not lysine, is actively secreted by RBCs infected by *P. falciparum*, and its synthesis may involve an important mechanism for removing and/or detoxifying excess lysine produced by fundamental haemoglobin degradation. Further studies are needed to control whether lysine degradation is essential in *P. falciparum*, which could be further explored as a potential novel drug target (34). Acetylation of lysine is a significant post-translational modification implicated in regulation of eukaryotic gene expression and other vital developments. This process can be hindered with inhibitors to his-tone de-acetylase which is now accepted as a confirmed strategy for cancer and infectious diseases which includes the asexual stage malaria parasites (35).

There have been many reports about the alteration in serum triacyl-glycerides during the course of malaria infection especially in children (36), but there are many reports are about TG change in the parasite. Parasites have the ability to make triacylglycerol from the mature trophozoite stage to the schizont stage and then in the later stages, this triacylglycerol is degraded into free fatty acids released into the medium during schizont rupture and/or merozoite discharge. This may describe the mechanism of action on host cells by malarial fatty acids (37).

There are reports of importance of haemozoin in different aspects of malaria, its crystallization is the target of widely used antimalarial aminoquinoline drugs. Host haemozoin production requires not only host lipids but also hydroxyl fatty acids generated by *Plasmodium* spp. in large amounts. Evidence has increasingly shown that hemozoin formation occurs most rapidly at lipid water interfaces. Neutral lipids were observed in the digestive vacuole of the parasite composed of di and triacylglycerols and seem to act as storage organelles for lipid intermediates which promote haemozoin formation (38).

## Conclusion

Since drugs targeting the sexual stages of *P. falciparum* are an essential pharmacological need for malaria elimination, the importance of eosin B gametocytocidal effect will be better understood. Eosin B is seen to have a notable gametocytocidal effect in vitro. The ^1^HNMR studies have shown that the differentiating metabolites are thiamine, L-aspartic acid, L-aspargine, L-tyrosine, L-lysine, NAD^+^, L-alanine, TG and phenylpyruvic acid which participate in the following six cycles including aminoacyl-tRNA biosynthesis; phenylalanine, tyrosine and tryptophan biosynthesis; alanine, aspartate and glutamate metabolism; phenylalanine metabolism, nicotinate and nicotinamide metabolism and lysine degradation. All these cycles and metabolites have been described as likely drug target effected by eosin B.

However, further work such as membrane feeding assay should be done to probe eosin B-mediated blocking of mosquito vector to human host malaria transmission.
